# Enhanced In Vivo Radiotherapy of Breast Cancer Using Gadolinium Oxide and Gold Hybrid Nanoparticles

**DOI:** 10.1021/acsabm.2c00965

**Published:** 2023-01-24

**Authors:** Hamed Nosrati, Marziyeh Salehiabar, Jalil Charmi, Kadir Yaray, Mohammadreza Ghaffarlou, Esra Balcioglu, Yavuz Nuri Ertas

**Affiliations:** † ERNAM—Nanotechnology Research and Application Center, Erciyes University, Kayseri39039, Türkiye; ‡ Department of Radiation Oncology, Faculty of Medicine, Erciyes University, Kayseri39039, Türkiye; § Department of Chemistry, Hacettepe University, Beytepe, Ankara06532, Türkiye; ∥ Department of Histology and Embryology, Faculty of Medicine, Erciyes University, Kayseri39039, Türkiye; ⊥ Department of Biomedical Engineering, Erciyes University, Kayseri39039, Türkiye; # UNAM−National Nanotechnology Research Center, Bilkent University, Ankara06800, Türkiye

**Keywords:** radiosensitizer, gadolinium, gold, nanoparticle, radiotherapy, bimetallic

## Abstract

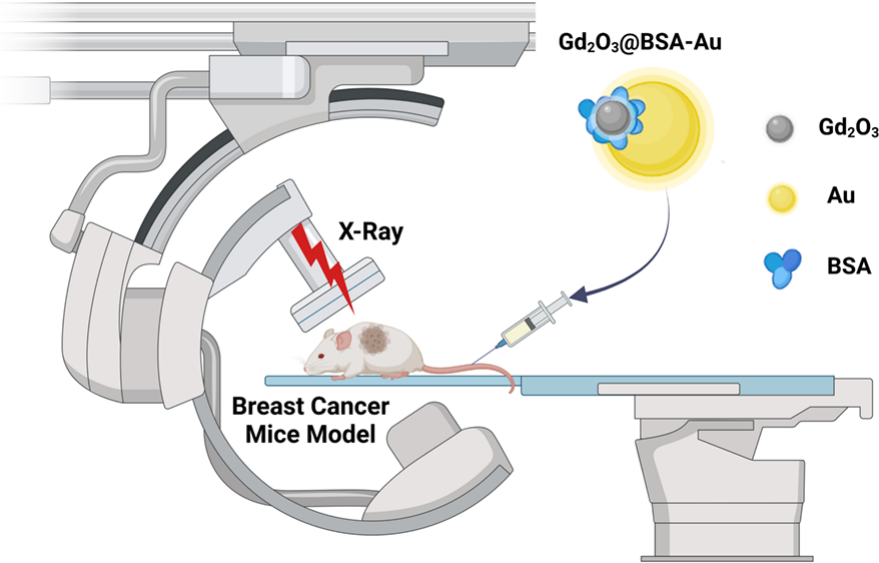

Radiation therapy has demonstrated promising effectiveness against several types of cancers. X-ray radiation therapy can be made further effective by utilizing nanoparticles of high-atomic-number (high-Z) materials that act as radiosensitizers. Here, in purpose of maximizing the radiation therapy within tumors, bovine serum albumin capped gadolinium oxide and gold nanoparticles (Gd_2_O_3_@BSA-Au NPs) are developed as a bimetallic radiosensitizer. In this study, we incorporate two high-Z-based nanoparticles, Au and Gd, in a single nanoplatform. The radiosensitizing ability of the nanoparticles was assessed with a series of in vitro tests, following evaluation in vivo in a breast cancer murine model. Enhanced tumor suppression is observed in the group that received radiation after administration of Gd_2_O_3_@BSA-Au NPs. As a result, cancer therapy efficacy is significantly improved by applying Gd_2_O_3_@BSA-Au NPs under X-ray irradiation, as evidenced by studies evaluating cell viability, proliferation, reactive oxygen species production, and in vivo anti-tumor effect.

## 1 Introduction

Radiotherapy is the use of high-energy X-rays in tumor regions to deliver irradiation doses for cancer treatment. Radiotherapy causes free radical damage or DNA damage to eradicate cancer cells.^1,2^ However, the therapeutic use of radiotherapy is constrained by its low radiosensitivity, inaccurate tumor localization, and poor differentiation between lesions and the adverse effects of irradiation in healthy tissues.^3^ Therefore, it is essential to find strategies to increase the radiosensitivity of tumors while simultaneously decreasing their systemic adverse effects. There is a widespread consensus that radiosensitization efforts should prioritize the integration of nanotechnology and radiation. In recent years, nanotechnology has been frequently used as a potential cancer detection and treatment technique in theranostics applications.^4−7^ Nanoparticles (NPs) have the capability of selectively accumulating in the tumor site through passive targeting, also known as the enhanced permeability and retention effect, which prolongs their circulation time.^8,9^ Our previous reports illustrated some of the beneficial properties of NPs favoring radiosensitizer agents in cancer treatment.^10−12^ NPs containing high atomic number (Z) elements, also known as heavy elements, such as gold (Au), gadolinium (Gd), and bismuth (Bi), have been shown as potential radiosensitizer agents due to their high X-ray photon capture cross section and Compton scattering effect.^13,14^ High Z-elements can release photoelectrons and Auger electrons under the X-ray irradiation which damage cancer cells through dose enhancement effect during radiation therapy.^15^ Due to their excellent absorption and ability to generate secondary electrons, AuNPs can boost X-ray dosage deposited in the tumor region and are considered potential radiation therapy sensitizers in cancer therapy.^16^ These properties of AuNPs led to the evaluation of their radiosensitizer role in different types of human cancers, including colon cancer,^17^ prostate cancer,^18^ cervix cancer,^19^ and breast cancer.^20^ Gadolinium-based NPs (GdNPs) with a high atomic number (64) have also been used as radiosensitizers.^21,22^ However, Gd, as a rare earth element of the lanthanide group, is inherently toxic.^23^ Therefore, different polymers such as hyaluronic acid (HA),^21,22^ bovine serum albumin (BSA),^24^ polyethylenimine (PEI) premodified with polyethylene glycol (PEG),^25,26^ and silica^27,28^ were used to coat GdNPs to passivate the surface of Gd and prevent toxicity. HA-Gd_2_O_3_ NPs were used in the MRI-guided radiotherapy of tumors where the NPs were found to display low cytotoxicity, excellent biocompatibility, and high hemocompatibility.^21^ Elsewhere, indocyanine green-loaded albumin–bioinspired gadolinium hybrid functionalized hollow gold nanoshells were produced as a nanotheranostic agent for photodynamic and photothermal therapy with near-infrared fluorescence imaging capability.^24^ Albumin-stabilized NPs enabled multiple modality imaging with excellent water dispersibility and biocompatibility. Hybrid Au/Gd_2_O_3_ NPs coated with PEI/PEG were developed as a dual mode magnetic resonance/computed tomography imaging agent, where cell viability tests displayed that HeLa cells maintained appropriate viability after treatment with the NPs at a concentration of 50 μM. It was demonstrated that not only PEI@Au/Gd_2_O_3_ NPs possessed excellent cytocompatibility but also Gd content in all organs had a similar trend to Au, indicating the excellent in vivo stability of the NPs.^25^

Herein, we synthesized BSA-coated Gd_2_O_3_ and Au NPs, Gd_2_O_3_@BSA-Au NPs, to increase the efficiency of radiotherapy by incorporating two high Z atoms, Au and Gd, into a single NP in order to minimize the local X-ray dose in tumors, hence reducing the side effects of radiotherapy.

## 2 Materials and Methods

### 2.1 Materials

Gadolinium(III) chloride hexahydrate (GdCl_3_·6H_2_O), BSA, tetrachloroauric(III) acid trihydrate (HAuCl_4_·3H_2_O), 3-(4,5-dimethylthiazol-2-yl)-2,5-diphenyltetrazolium bromide (MTT), 2′,7′-dichlorodihydrofluorescein diacetate (DCFH-DA), trisodium citrate, and sodium hydroxide (NaOH) were purchased from Sigma-Aldrich (USA). Crystal violet was supplied from AFG Bioscience (USA). Calcein-acetoxymethyl (Calcein-AM) and propidium iodide (PI) were obtained from AAT Bioquest (USA). All materials were used without further purification.

### 2.2 Methods

#### 2.2.1 Synthesis of Gd_2_O_3_@BSA NPs

**Figure 1:**
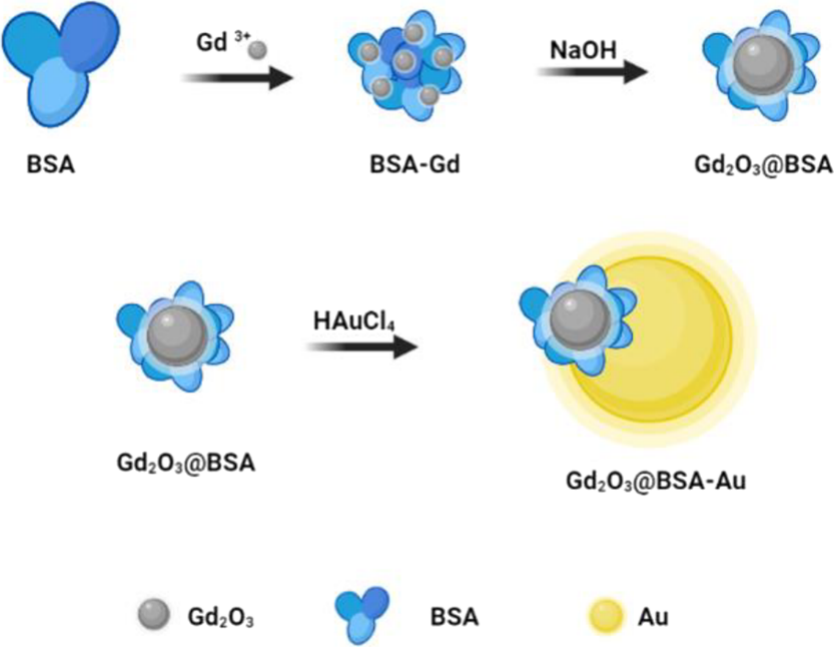
Schematic illustration of the synthesis process of Gd_2_O_3_@BSA and Gd_2_O_3_@BSA-Au nanoparticles.

GdCl_3_·6H_2_O solution (100 mM, 1.0 mL) was added to BSA solution (9.0 mL, 250 mg) under vigorous stirring. Following the adjustment of the pH to 12 with NaOH (2.0 M), the reaction was stirred overnight at 37 °C. To obtain Gd_2_O_3_@BSA, the mixture was dialyzed against distilled water for a further 24 h to eliminate any excess precursors. The synthesis process is shown in Figure 1.

#### 2.2.2 Synthesis of Gd_2_O_3_@BSA-Au NPs

By growing Au NPs on the surface of Gd_2_O_3_@BSA in situ, Gd_2_O_3_@BSA-Au heterojunction NPs were synthesized. Briefly, deionized water (50 mL) containing Gd_2_O_3_@BSA (30 mg) was heated to 120 °C. Next, HAuCl_4_·3H_2_O (15 mg) was added to the solution. Subsequently, trisodium citrate (65 mg) in deionized water (1.5 mL) was added to the reaction medium. After performing the reaction for 30 min and cooling down to room temperature, the solution was purified with the dialysis process against deionized water. Schematic illustration of the synthesis method is shown in Figure 1.

### 2.3 Characterization

The crystalline structure of the various samples was characterized by X-ray diffraction (XRD; X-ray diffractometer, Malvern Panalytical Empyrean) by using a Cu-Kα1 radiation source, λ = 0.15406 nm. The XRD data in 2θ ranging from 20 to 80° were collected with a scanning step size of 0.02°. Elemental mapping of nanostructures was determined by transition electron microscopy (TEM; FEI Tecnai at 120 keV). Field emission scanning electron microscopy (FE-SEM; ZEISS, GEMINI 500) was used to determine the morphology and size of the NPs. Dynamic light scattering (DLS; Malvern Instruments, Nano ZS90) was used to evaluate the hydrodynamic diameter and surface charge (ζ) of particles. To understand the physical and chemical properties of different samples, ultraviolet–visible (UV–Vis; Perkin Elmer, Lambda 25) and Fourier-transform infrared spectroscopy (FT-IR; Shimadzu, IRTracer-100) were used. X-ray photoelectron experiments were performed using a mono-chromatized Al-Kα X-ray source (Thermo Scientific).

### 2.4 Hemolysis Assay

Hemolytic activity is a requirement to be tested for any blood in contact with NPs. Because NPs come into contact with blood in the biological environment, it is critical to identify whether or not the NPs are blood compatible, which is assessed by the hemolysis test. For this purpose, a solution (500 μL) of human red blood cells containing NPs at different concentrations was poured into Eppendorf tubes and placed in a shaker at 37 °C for 4 h. Phosphate-buffered saline (PBS) and distilled water were used as the negative (0% lysis) and positive controls (100% lysis), respectively. After centrifugation at 3000 rpm for 10 min, the supernatant’s absorbance was measured at 540 nm. The hemolysis percentage was calculated using the following equation.
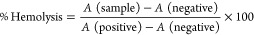
1

### 2.5 Cytotoxicity Assay

The cytotoxicity of Gd_2_O_3_@BSA-Au NPs was determined by applying an MTT assay on human umbilical vein endothelial cells (HUVECs). Briefly, 5000 cells in the complete culture medium (100 μL) were transferred to a 96-well plate and incubated at 37 °C for 24 h. To evaluate the biocompatibility of Gd_2_O_3_@BSA-Au NPs, the medium was replaced with a fresh medium containing Gd_2_O_3_@BSA-Au NPs at various concentrations (10, 20, 40, and 80 μg/mL) and then incubated for 5 h. The previous culture medium was refreshed with a culture medium in each related well. In the following step, after 24 h of incubation (37 °C and moisture containing 5% CO_2_), MTT (20 μL, 5 mg/mL) was added to each well and the plate was incubated for further 4 h. To dissolve the generated formazan, dimethyl sulfoxide (100 μL) was added to each well following discarding the medium. Finally, to measure the cell viability, the absorbance was recorded at a wavelength of 570/640 nm with a microplate reader (BioTek Synergy HTX). To determine the anticancer effect of Gd_2_O_3_@BSA-Au NPs in the presence and absence of X-ray irradiation, MTT assay was used in the same way mentioned where X-ray irradiation was performed at a dose of 5 Gy (6 MV on the 4T1 cell line).

### 2.6 Calcein AM/PI Staining

Live and dead cells, after different treatments, were imaged following staining with Calcein AM and PI. In a 96-well plate, 5 × 10^3^ 4T1 cells per well were seeded, and after incubation for 24 h, the cells were treated with NPs for further 5 h. There were five experimental groups: PBS, X-ray, Gd_2_O_3_@BSA + X-ray, Gd_2_O_3_@BSA-Au, and Gd_2_O_3_@BSA-Au + X-ray. After exposing to X-ray irradiation, the cells were incubated for further 24 h. The cells were co-stained with Calcein AM (100 μL, 3 μM) and PI (100 μL, 4 μM) for 30 and 5 min, respectively. Finally, the treated plate was evaluated by fluorescence microscopy (Leica DM IL LED Fluo).

### 2.7 Reactive Oxygen Species (ROS) Assay

2′,7′-Dichlorofluorescin diacetate (DCFH-DA), a cell-permeant reagent fluorogenic dye that assesses hydroxyl, peroxyl, and other ROS activities in the cell, was used to quantify intracellular ROS formation. Following cellular uptake, DCFH-DA is deacetylated by cellular esterases to a nonfluorescent molecule, which is then oxidized by ROS to produce green fluorescence. To assess the capacity of designed NPs to produce ROS, 1 × 10^3^ 4T1 cells per well were seeded in a 96-well plate and incubated overnight. The prepared samples including Gd_2_O_3_@BSA and Gd_2_O_3_@BSA-Au with and without X-ray irradiation were evaluated by DCFH-DA which shows a green fluorescence image in the presence of ROS production. Prepared groups including control PBS, X-ray, Gd_2_O_3_@BSA + X-ray, Gd_2_O_3_@BSA-Au, and Gd_2_O_3_@BSA-Au + X-ray were co-incubated for further 5 h. Then, DCFH-DA (100 μL, 10 μM) was added to each well and incubated for 1 h. Related groups to the radiotherapy were irradiated (5 Gy and 6 MV). Then, the samples were imaged by fluorescence microscopy.

### 2.8 Colony Formation Assay

The clonogenic assay, also called the colony formation assay, is a test of a single cell’s ability to grow into a group of cells.^29^ The rate of growth in different treatment groups provides visual results about inhibition or growth of the cancer cell line into the colony. It means that the generated colony decreases with the treatment element in this assay. In detail, 4T1 cells (300 cells/well) were seeded in a 6-well plate in a medium cell culture and incubated for 24 h. The cells were incubated for 7 days after treatment and then washed with PBS, and a mixture of methanol and acetic acid (3:1) was used to fix the cells. After 5 min, the cells were stained with 0.5% crystal violet solution in methanol. In the next following step, the content of each well was washed with deionized water after 15 min, and the number of colonies was counted using Image J software. The cell survival fraction was calculated using the following formulas:
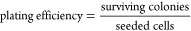
2
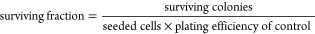
3

### 2.9 In Vivo Anticancer Study

To evaluate the radiosensitizer efficiency of NPs in the animal model, 4T1 cells (1 × 10^6^) were subcutaneously injected into the right flank of Balb/C mice to generate the 4T1 murine mammary carcinoma tumor. When the tumor volume reached 200 mm^3^, treatment was started. Tumor volume was calculated according to the following equation.
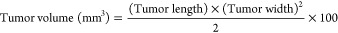


Then, five groups including control, X-ray, Gd_2_O_3_@BSA + X-ray, Gd_2_O_3_@BSA-Au, and Gd_2_O_3_@BSA-Au + X-ray with five mice in each group were provided. After 24 h of intravenous injection, related groups were exposed to radiation (5 Gy, 6 MV) for the treatment. Tumor size and weight of mice were monitored in different days to observe changes in the treatment process (20 days).

## 3 Results and Discussion

### 3.1 Synthesis and Characterization

**Figure 2:**
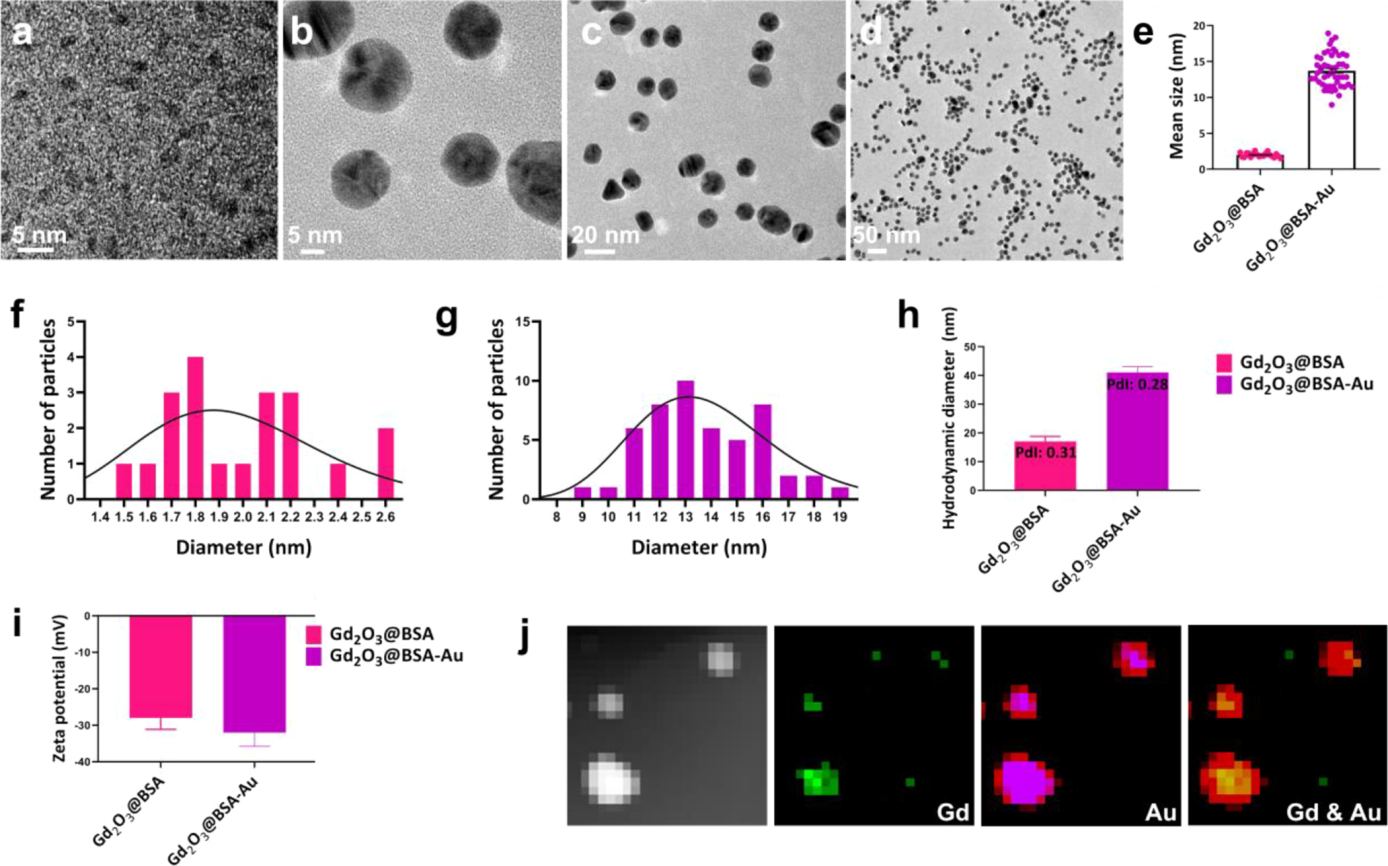
TEM image of (a) Gd_2_O_3_@BSA and (b–d) Gd_2_O_3_@BSA-Au; corresponding size distribution histograms with a lognormal fit (e) Gd_2_O_3_@BSA and Gd_2_O_3_@BSA-Au; (f–g) diameter distribution for Gd_2_O_3_@BSA and Gd_2_O_3_@BSA-Au; (h) hydrodynamic size of NPs; (i) zeta potential of NPs; (j) TEM-EDS mapping of Gd_2_O_3_@BSA-Au.

Gd_2_O_3_ nanocrystals were synthesized within a BSA nanoreactor’s expanding cavity.^30^ Albumin’s abundance of active groups such as sulfhydryl and carboxyl groups may cause the formation of metal ion complexes.^31,32^ Finally, at a pH of 12, the unfolding process of albumin was triggered by NaOH, leading to its expansion, and the nucleation of Gd_2_O_3_ under precipitation reaction.^30,33^ As a result, Gd_2_O_3_@BSA nanocrystals were synthesized with average diameters of 2 ± 0.3 nm (Figure 2a). For preparation of Gd_2_O_3_@BSA-Au, HAuCl_4_ was reduced in the presence of Gd_2_O_3_@BSA via a Turkevich method. Gd_2_O_3_@BSA-Au had a mean dimension of 13.7 ± 2.3 nm. Gd_2_O_3_@BSA-Au structure contained two different parts, darker and bright (Figure 2b,c), where the regions Gd_2_O_3_ and Au NPs overlapped look darker, due to rise in electron densities. Figure 2d clearly shows the monodispersity of Gd_2_O_3_@BSA-Au. It was also found that Gd_2_O_3_@BSA-Au had a uniform spherical shape. Figure 2e shows average diameters of Gd_2_O_3_@BSA and Gd_2_O_3_@BSA-Au. Size distributions of Gd_2_O_3_@BSA and Gd_2_O_3_@BSA-Au are also shown in Figure 2f,g. The hydrodynamic sizes of Gd_2_O_3_@BSA and Gd_2_O_3_@BSA-Au were measured to be 17 and 41 nm, respectively, and the zeta potentials were found to be −28 and −32 mV, respectively (Figure 2h,i). The stability of Gd_2_O_3_@BSA-Au was studied by size monitoring; it was found that the hydrodynamic size did not change significantly for 30 days (Figure S1a). Figure S1b,c shows the SEM-EDS spectrum and mapping of Gd_2_O_3_@BSA-Au for elemental analysis of the binary system, where both data confirm the existence of Gd, Au, and C as the main elements of NPs. The assigned Gd and Au phase identities are further corroborated by elemental maps produced through TEM (Figure 2j).

**Figure 3:**
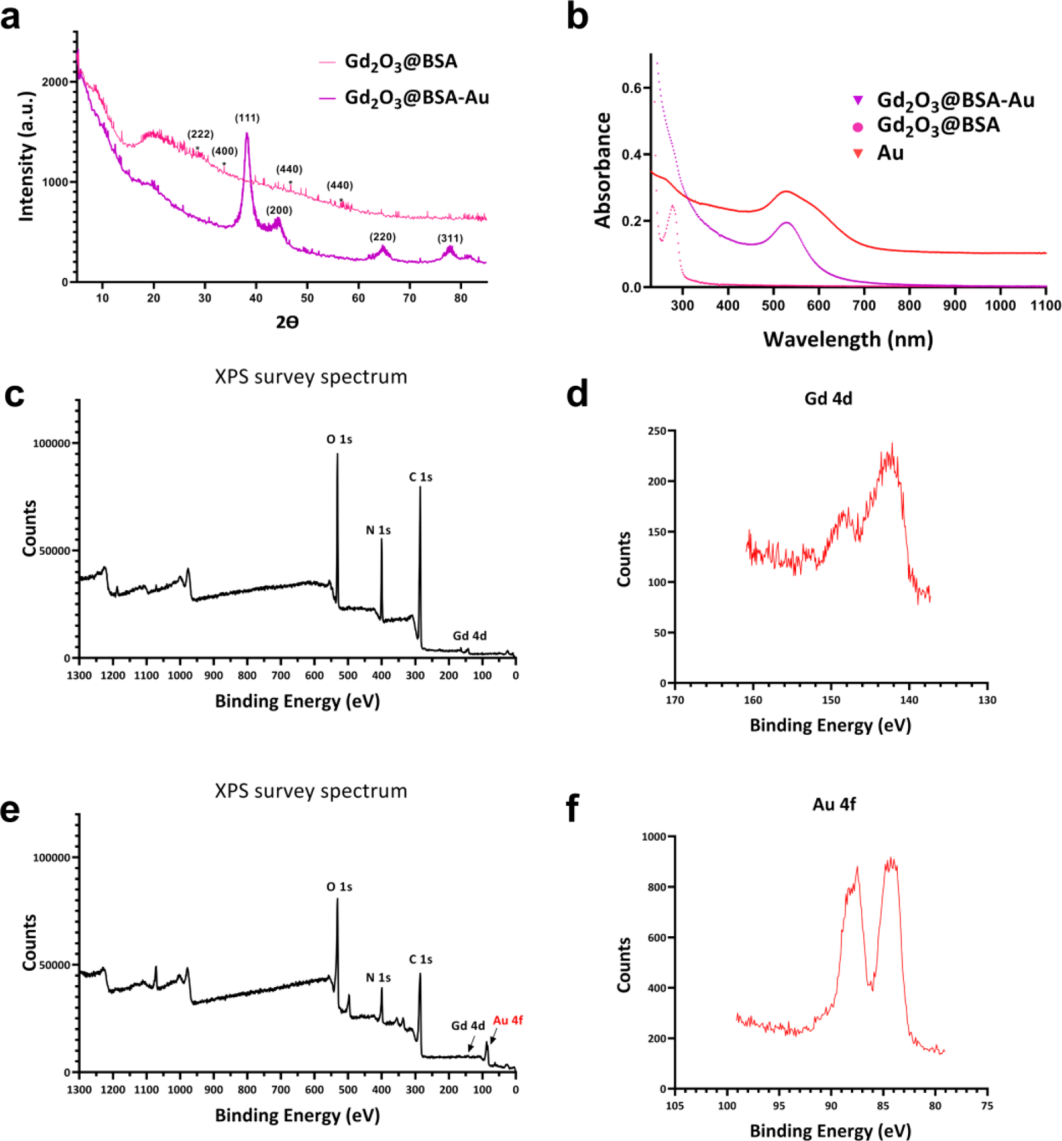
(a) XRD patterns of Gd_2_O_3_@BSA and Gd_2_O_3_@BSA-Au; (b) UV–Vis spectra of Au, Gd_2_O_3_@BSA, and Gd_2_O_3_@BSA-Au; (c) XPS survey wide scans of Gd_2_O_3_@BSA; (d) Gd 4d XPS spectra in Gd_2_O_3_@BSA; (e) XPS survey wide scans of Gd_2_O_3_@BSA-Au; (f) Au 4f XPS spectra in Gd_2_O_3_@BSA-Au.

The XRD patterns of Gd_2_O_3_@BSA and Gd_2_O_3_@BSA-Au are shown in Figure 3a, where the broad peak at 2θ = ∼20° corresponds to the BSA shell. Because the amount of BSA is higher than that of Gd, the peak intensities of XRD diffraction peaks of Gd_2_O_3_ are lower and covered within the BSA shell, whereas XRD diffraction peaks of Au are sharper and clearly detected.

Furthermore, UV–Vis spectrophotometry was used to study the optical property of NPs. UV–Vis spectra of Au, Gd_2_O_3_@BSA, and Gd_2_O_3_@BSA-Au NPs are shown in Figure 3b. Au NPs show the typical surface plasmon resonance (SPR) band at 525 nm,^34^ while no such peak is present for Gd_2_O_3_@BSA. Gd_2_O_3_@BSA-Au NPs not only display a typical SPR band of Au at 528 nm but also show the characteristic absorbance of BSA at 271 nm like a shoulder.

X-ray photoelectron spectroscopy (XPS) was used to examine the chemical state and components of Gd_2_O_3_@BSA and Gd_2_O_3_@BSA-Au to confirm the successful synthesis. The XPS survey spectrum of Gd_2_O_3_@BSA shows the Gd 4d, C 1s, N 1s, and O 1s peaks (Figure 3c). The Gd 4d_5/2_ and Gd 4d_3/2_ spin–orbits were responsible for the binding energy maxima at 142.4 and 148.5 eV, respectively, in the high resolution Gd 4d XPS spectrum (Figure 3d).^35^ Moreover, the XPS wide spectrum of Gd_2_O_3_@BSA-Au shows the Gd 4d, C 1s, N 1s, and O 1s peaks along with the Au 4f peak (Figure 3e). The high-resolution Gd 4d XPS spectrum of Gd_2_O_3_@BSA-Au shows Gd 4d_5/2_ and Gd 4d_3/2_ spin–orbit peaks at the binding energies of 141.7 and 148.01 eV, respectively (Figure S2b).^36^ In the 4f high-resolution spectrum of gold, there are two peaks that correspond to the 4f_7/2_ and 4f_5/2_ of Au (0), respectively, with binding energies of 84.3 and 87.9 eV (Figure 3f).^37,38^ The presence of C 1s peaks confirms the existence of BSA in the structure of Gd_2_O_3_@BSA and Gd_2_O_3_@BSA-Au NPs (Figure S2a,c).

### 3.2 Hemocompatibility

**Figure 4:**
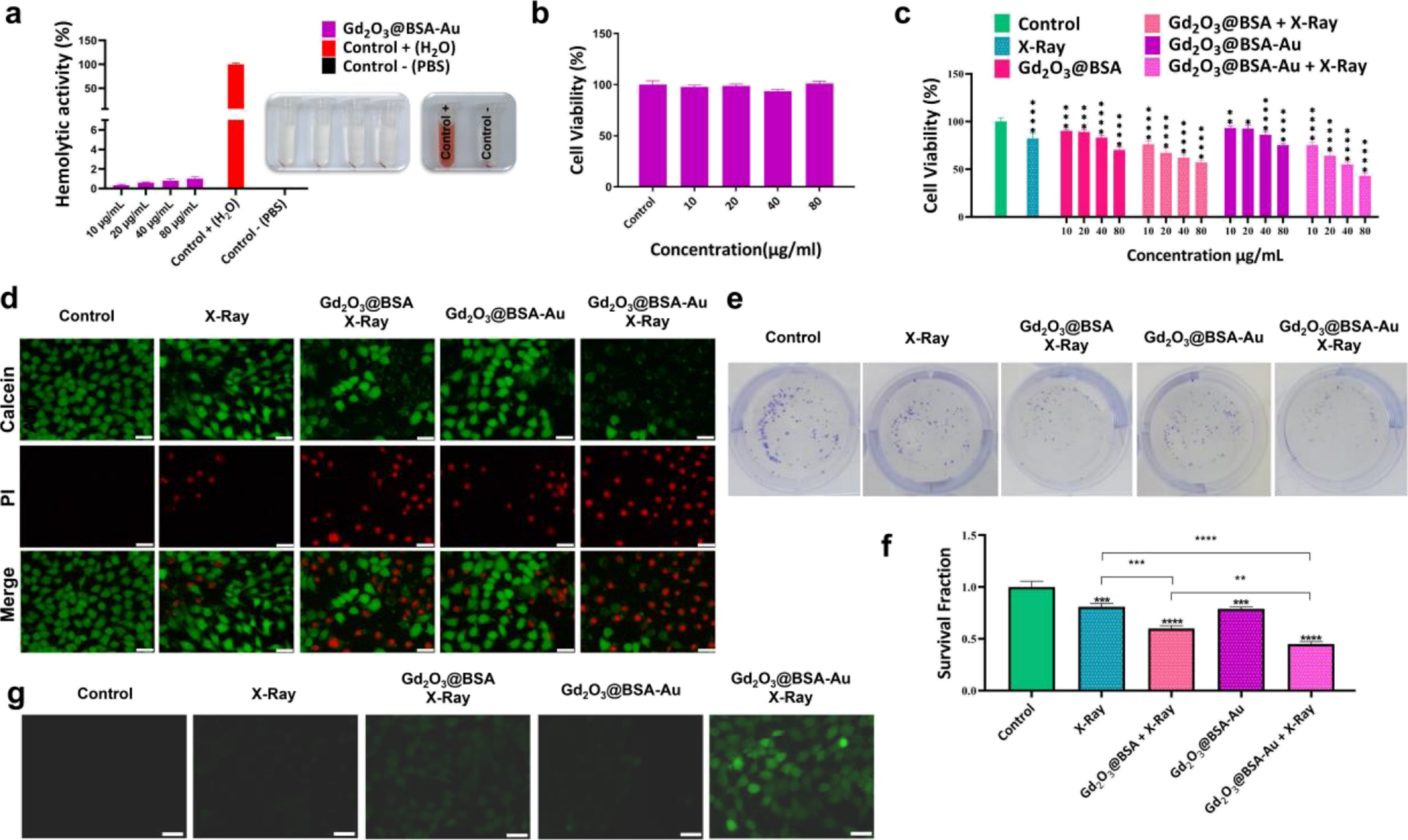
(a) Hemolysis assay graph and related photographs; (b) cell viability result of Gd_2_O_3_@BSA-Au on HUVEC cells; in vitro anticancer result of different treatment plans in the presence and absence of X-ray irradiation. (c) MTT assay on 4T1 cells, (d) imaging of live and dead cells stained with Calcein AM/PI, (e) digital photographs of the clonogenic assay; (f) cell survival data obtained from the clonogenic assay; (g) intracellular ROS production detection in the presence and absence of X-ray irradiation. Data = mean ± SD; *p < 0.05, **p < 0.01, ***p < 0.001, and ****p < 0.0001 compared to the control group.

An ideal formulation for intravenous injection should be biocompatible with blood components. As a consequence, the hemolytic activity of Gd_2_O_3_@BSA-Au at various concentrations (0, 10, 20, 40, and 80 μg/mL) was examined, and the lysis profiles of red blood cells (RBCs) were expressed as a percentage of hemoglobin released in contrast to the positive and negative controls. The concentration of released hemoglobin when blood is exposed to NPs was used to calculate the proportion of NP-induced hemolysis. Figure 4a displays images of RBCs taken after they had been exposed to the NPs. Figure 4a shows that Gd_2_O_3_@BSA-Au NPs did not cause any obvious hemolysis. On the other hand, Gd_2_O_3_@BSA-Au showed no significant hemolytic activity, even at the highest dose tested (80 μg/mL), indicating high safety for intravenous administration. Damage to red blood cells occurs when the hemolysis value is more than 5%, as required by the ASTM E2524-08 standard.^39^ It is believed that biocompatible hydrophilic coating can reduce the hemolytic potential of NPs, which would prevent the NPs from being detected as foreign objects by biological cells.^40,41^

### 3.3 Cytotoxicity

Using the MTT test, the biocompatibility of Gd_2_O_3_@BSA-Au was assessed at the cellular level. There was no significant cytotoxicity when HUVEC cells were incubated with Gd_2_O_3_@BSA-Au even at a concentration of 80 μg/mL, which shows the biocompatibility of Gd_2_O_3_@BSA-Au NPs (Figure 4b).

In order to evaluate the anticancer effect of Gd_2_O_3_@BSA-Au with or without X-ray irradiation, the MTT test was applied as well. Gd_2_O_3_@BSA and Gd_2_O_3_@BSA-Au showed dose-dependent cytotoxicity (Figure 4c). The same manner was also observed under X-ray irradiation. Therefore, when cells were exposed to X-rays (5 Gy), their viability went down as the concentration increased from 10 to 80 μg/mL. When cells were treated with Gd_2_O_3_@BSA and Gd_2_O_3_@BSA-Au at a concentration of 40 μg/mL without X-ray irradiation, their viability was still around 81 and 86%, respectively. However, it dropped to 62 and 55% when exposed to X-ray irradiation, respectively, for Gd_2_O_3_@BSA and Gd_2_O_3_@BSA-Au (Figure 4c). Accordingly, IC_50_ values were found to be 245.8 and 97.2 μg/mL for Gd_2_O_3_@BSA + X-ray and Gd_2_O_3_@BSA-Au + X-ray treatment groups, respectively.

The Calcein-AM/PI cell staining assay was used to assess the ability of NPs to cause cell death. Calcein-AM and PI solutions are employed in this approach to stain live and dead cells, respectively. Live and dead 4T1 cells after application of different treatments were imaged following staining with dyes (Figure 4d). Calcein-AM dye stains the live cells in green, while PI was used to mark the dead cells in red.^42^ The growing intensity of red colored dots signifies a higher rate of cell death, whereas the green color reflects live cells. In the control group, only green fluorescence was observed, and it is obvious that X-ray irradiation alone had little killing effect (little red spots detected) on 4T1 cells in the absence of NP treatment. For the cells treated with X-rays only or Gd_2_O_3_@BSA-Au, green fluorescence dominated along with a small amount of red fluorescence. Co-treatment of 4T1 cells with X-rays and NPs, on the other hand, resulted in increased cell death, depicted by high levels of red fluorescence intensity. The highest red and lowest green fluorescence intensities were observed for the Gd_2_O_3_@BSA-Au + X-ray group, indicating the high anticancer efficacy. This is owing to the usage of high-Z metals in the structure of NPs, such as Gd and Au, which results in the formation of more oxygen-sensitive species in the presence of X-ray irradiation.

### 3.4 Colony Formation Assay

An in vitro cell survival test, clonogenic assay, which is widely used to detect cell reproductive mortality following irradiation, was performed to determine the level of cell survival and clonogenicity (Figure 4e). To facilitate the statistical analysis of clonogenic assay, survival data were plotted as bar graphs (Figure 4f). When compared to the control group, colony formation was significantly reduced in cells treated with X-rays alone and all of the NP groups in combination with X-rays. Compared to the untreated groups, the addition of NPs (Gd_2_O_3_@BSA or Gd_2_O_3_@BSA-Au) increased radiation-induced cell death. Noteworthy is the fact that the NP-induced radiosensitization of Gd_2_O_3_@BSA-Au was higher than that of Gd_2_O_3_@BSA because of the presence of two high-Z elements in one system.^38^ This finding adds to the evidence that high-Z metals can be used to make nanoradiosensitizers that generate effective ROS products in the presence of X-ray irradiation, thereby limiting cancer cell repopulation.

### 3.5 ROS Generation Assay

Radiation-induced DNA damage caused by X-rays increases the efficacy of cancer treatment. DCFH-DA, a dye that can be oxidized to emit bright green fluorescence, was used as a probe to measure intracellular ROS generation (Figure 4g).^43^ The control group, which was not exposed to X-rays or NPs, displayed no green fluorescence, suggesting that no ROS were produced. However, for the cells treated with X-ray irradiation only, slight green fluorescence was observed. As expected, the cells treated with Gd_2_O_3_@BSA or Gd_2_O_3_@BSA-Au under X-ray irradiation demonstrated the strongest green fluorescence. The green fluorescence intensity in the Gd_2_O_3_@BSA-Au + X-ray group was much higher than that of Gd_2_O_3_@BSA + X-ray. Also, the cells treated with Gd_2_O_3_@BSA-Au only without any irradiation expressed limited green fluorescence. Therefore, the application of X-rays significantly increases the ROS production and bimetallic Gd_2_O_3_@BSA-Au NPs have a higher ROS generation enhancing ability than Gd_2_O_3_@BSA. NPs containing high Z-elements, under the X-ray irradiation, can release of secondary electrons, resulting in enhanced ROS production within cells.^10,44^

### 3.6 In Vivo Anticancer Study

**Figure 5:**
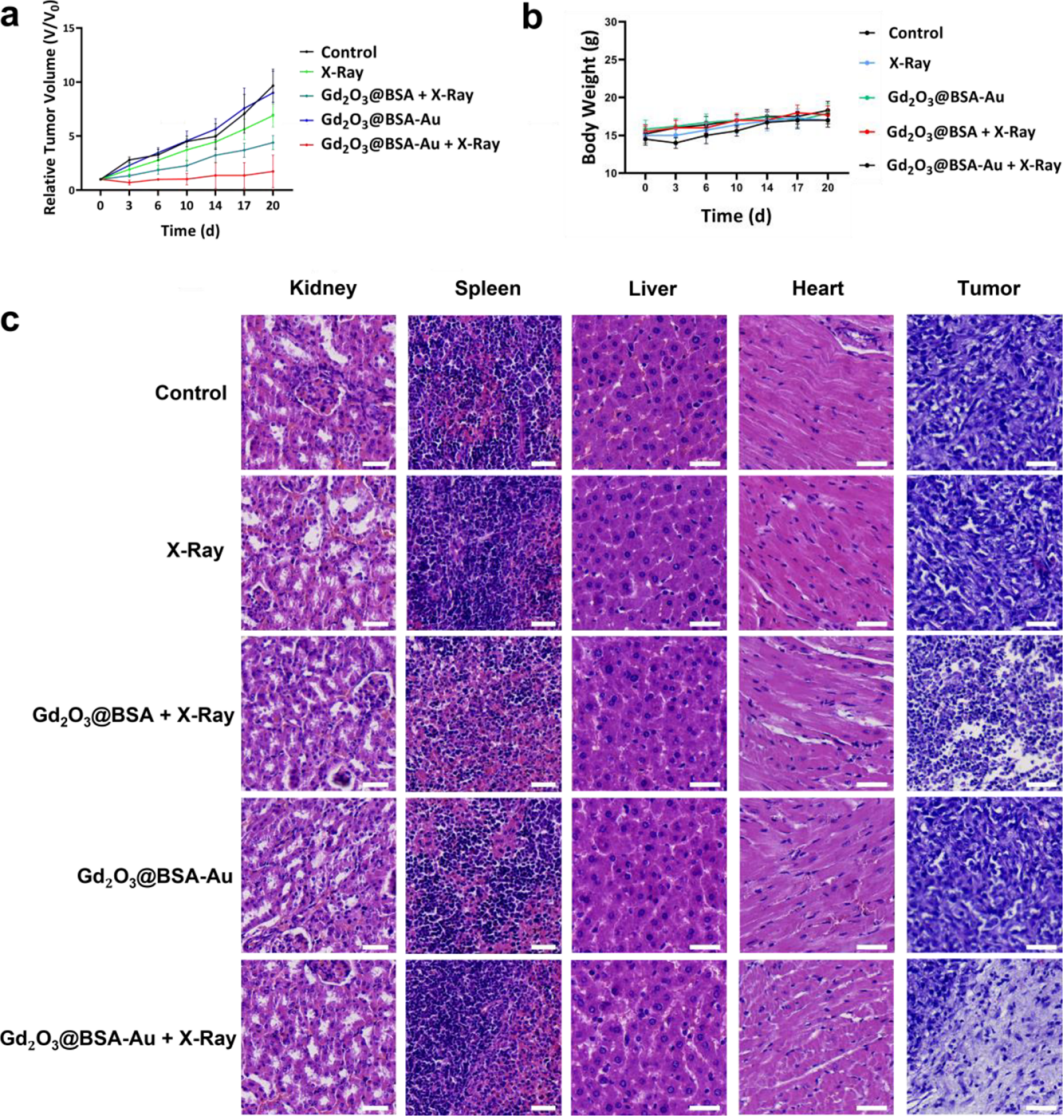
Histological analysis of the key organs of mice treated with PBS and Gd_2_O_3_@BSA-Au; (a) relative tumor volume of mice treated with various treatment plans; (b) body weight of treated mice; (c) histological analysis of the tumors of mice treated with various treatment plans. Scale bars: 50 μm.

Based on the in vitro assay results, we investigated the anticancer effects of experimental groups in vivo. The radioenhancing abilities were conducted in 4T1 tumor-bearing mice. When the mean tumor volume reached 200 mm^3^, mice were randomly divided into five groups of five mice each and treatment was started. The mice were exposed to X-ray irradiation (5 Gy and 6 MV), 24 h after injection of NPs. In vivo tumor reduction results following intravenous administration are shown in Figure 5a. The control group did not display tumor growth inhibition effect, and Gd_2_O_3_@BSA-Au without X-rays was also ineffective in suppressing tumor development. Application of X-rays alone had a slightly better but still poor anticancer activity. However, combination of intravenous injection of NPs with X-rays resulted in substantially altered effect. The group treated with Gd_2_O_3_@BSA + X-ray showed tumor growth inhibitory effect, but the highest tumor growth inhibitory effect appeared when mice were treated with Gd_2_O_3_@BSA-Au + X-ray. Within 14 days following a single intravenous injection of Gd_2_O_3_@BSA-Au paired with X-ray radiation, three mice out of five were completely tumor-free. Furthermore, any changes in the body weight of the mice were thoroughly tracked across the experimental time periods, where no significant changes in body weight were found across all experimental groups, highlighting the utility of Gd_2_O_3_@BSA-Au NPs as a safe radiosensitizer in cancer treatment (Figure 5b).^43^

In vivo treatment efficacy was also monitored with hematoxylin & eosin (H&E) staining of healthy and tumor tissues to evaluate the tissue damage to tumor cells and possible side effects to healthy organs (Figure 5c). The main organs such as the kidneys, spleen, liver, and heart with various treatments indicated no obvious tissue damage, indicating the biocompatibility of NPs for use as in vivo breast cancer therapy. The tumor cellular status of the control, X-ray, and Gd_2_O_3_@BSA-Au groups did not show clear changes, whereas Gd_2_O_3_@BSA + X-ray treatment resulted in moderate damage to tumor tissue. However, large shadow areas and necrotic-shaped cells were observed in Gd_2_O_3_@BSA-Au + X-ray groups, which confirm the effective destruction of tumor cells.^10^ Here, the presence of Au NPs could further enhance ROS levels and kill tumors by blocking cell proliferation.^45^

## 4 Conclusions

Nanosensitizers can promote the generation of ROS within cancer cells and kill them via various mechanisms. Here, bimetallic Gd_2_O_3_@BSA-Au NPs were developed and evaluated as a radiosensitizer under X-ray irradiation. NP dose-dependent radioenhancement effect was observed, where a high level of ROS was generated within cancer cells, and cancer cell death was induced under single X-ray irradiation, which was confirmed with in vitro biological assays. In vivo tumor treatment efficacy was further assessed in the murine model. When nanohybrid metallic particles were applied in conjunction with X-ray irradiation, inhibition of tumor growth was documented compared to control groups. Biosafety tests revealed no negative effects on healthy organs, confirming the safe use of the developed NPs in preclinical and clinical stages. Overall, the utilization of high atomic number bimetallic nanoradiosensitizers containing gadolinium and gold was shown to be highly effective toward cancer radiotherapy. Considering the current clinical application of radiotherapy consisting of repeated sessions of irradiations for several months, NPs may offer enhanced treatment outcome with a single X-ray irradiation as demonstrated in this work both in vitro and in vivo.

## Supporting Information Available

Size monitoring of Gd_2_O_3_@BSA-Au NPs in PBS, EDS spectra of Gd_2_O_3_@BSA-Au NPs, SEM-EDS mapping of Gd_2_O_3_@BSA-Au NPs, C 1S XPS spectra of Gd_2_O_3_@BSA, Gd 4d XPS spectra of Gd_2_O_3_@BSA-Au, and C 1S XPS spectra of Gd_2_O_3_@BSA-Au (PDF)

## Notes

This study was approved by the Ethics Committee of the Erciyes University (#2022/262).

## Notes

The raw/processed data required to reproduce these findings cannot be shared at this time as the data also form part of an ongoing study.

## References

[ref1] Lord C. J.; Ashworth A. The DNA damage response and cancer therapy. Nature 2012, 481, 287–294. 10.1038/nature10760. 22258607

[ref2] Huang Y.; Luo Y.; Zheng W.; Chen T. Rational design of cancer-targeted BSA protein nanoparticles as radiosensitizer to overcome cancer radioresistance. ACS Appl. Mater. Interfaces 2014, 6, 19217–19228. 10.1021/am505246w. 25314331

[ref3] Xing H.; Zheng X.; Ren Q.; Bu W.; Ge W.; Xiao Q.; Zhang S.; Wei C.; Qu H.; Wang Z. Computed tomography imaging-guided radiotherapy by targeting upconversion nanocubes with significant imaging and radiosensitization enhancements. Sci. Rep. 2013, 3, 1751 10.1038/srep01751. 23624542 PMC3638198

[ref4] Luo Z.; Ding X.; Hu Y.; Wu S.; Xiang Y.; Zeng Y.; Zhang B.; Yan H.; Zhang H.; Zhu L.; Liu J.; Li J.; Cai K.; Zhao Y. Engineering a hollow nanocontainer platform with multifunctional molecular machines for tumor-targeted therapy in vitro and in vivo. ACS Nano 2013, 7, 10271–10284. 10.1021/nn404676w. 24127723

[ref5] Nosrati H.; Salehiabar M.; Mozafari F.; Charmi J.; Erdoğan N.; Ghaffarlou M.; Abhari F.; Danafar H.; Ramazani A.; Nuri Ertas Y. Preparation and evaluation of bismuth sulfide and magnetite-based theranostic nanohybrid as drug carrier and dual MRI/CT contrast agent. Appl. Organomet. Chem. 2022, 36, e6861 10.1002/aoc.6861.

[ref6] Ashrafizadeh M.; Zarrabi A.; Karimi-Maleh H.; Taheriazam A.; Mirzaei S.; Hashemi M.; Hushmandi K.; Makvandi P.; Nazarzadeh Zare E.; Sharifi E.; et al. (Nano)platforms in bladder cancer therapy: Challenges and opportunities. Bioeng. Transl. Med. 2023, 8, e10353 10.1002/btm2.10353. 36684065 PMC9842064

[ref7] Wei W. J.; Zarghami N.; Abasi M.; Ertas Y. N.; Pilehvar Y. Implantable magnetic nanofibers with ON-OFF switchable release of curcumin for possible local hyperthermic chemotherapy of melanoma. J. Biomed. Mater. Res. A 2022, 110, 851–860. 10.1002/jbm.a.37333. 34786813

[ref8] Liao Y.-T.; Liu C.-H.; Yu J.; Wu K. C. Liver cancer cells: targeting and prolonged-release drug carriers consisting of mesoporous silica nanoparticles and alginate microspheres. Int. J. Nanomed. 2014, 9, 2767 10.2147/IJN.S60171. PMC4051719 24940057

[ref9] Jan N.; Madni A.; Khan S.; Shah H.; Akram F.; Khan A.; Ertas D.; Bostanudin M. F.; Contag C. H.; Ashammakhi N.; et al. Biomimetic cell membrane-coated poly(lactic-co-glycolic acid) nanoparticles for biomedical applications. Bioeng. Transl. Med. 2022, e10441 10.1002/btm2.10441. PMC10013795 36925703

[ref10] Abhari F.; Charmi J.; Rezaeejam H.; Karimimoghaddam Z.; Nosrati H.; Danafar H.; Farajollahi A. Folic Acid Modified Bismuth Sulfide and Gold Heterodimers for Enhancing Radiosensitization of Mice Tumors to X-ray Radiation. ACS Sustainable Chem. Eng. 2020, 8, 5260–5269. 10.1021/acssuschemeng.0c00182.

[ref11] Nosrati H.; Charmi J.; Abhari F.; Attari E.; Bochani S.; Johari B.; Rezaeejam H.; Manjili H. K.; Davaran S.; Danafar H. Improved synergic therapeutic effects of chemoradiation therapy with the aid of a co-drug-loaded nano-radiosensitizer under conventional-dose X-ray irradiation. Biomater. Sci. 2020, 8, 4275–4286. 10.1039/D0BM00353K. 32589170

[ref12] Salehiabar M.; Ghaffarlou M.; Mohammadi A.; Mousazadeh N.; Rahimi H.; Abhari F.; Rashidzadeh H.; Nasehi L.; Rezaeejam H.; Barsbay M.; Ertas Y. N.; Nosrati H.; Kavetskyy T.; Danafar H. Targeted CuFe2O4 hybrid nanoradiosensitizers for synchronous chemoradiotherapy. J. Controlled Release 2023, 353, 850–863. 10.1016/j.jconrel.2022.12.004. 36493951

[ref13] Dufort S.; Bianchi A.; Henry M.; Lux F.; Le Duc G.; Josserand V.; Louis C.; Perriat P.; Crémillieux Y.; Tillement O. Nebulized gadolinium-based nanoparticles: a theranostic approach for lung tumor imaging and radiosensitization. Small 2015, 11, 215–221. 10.1002/smll.201401284. 25201285

[ref14] Ma M.; Huang Y.; Chen H.; Jia X.; Wang S.; Wang Z.; Shi J. Bi2S3-embedded mesoporous silica nanoparticles for efficient drug delivery and interstitial radiotherapy sensitization. Biomaterials 2015, 37, 447–455. 10.1016/j.biomaterials.2014.10.001. 25453972

[ref15] Hossain M.; Su M. Nanoparticle location and material-dependent dose enhancement in X-ray radiation therapy. J. Phys. Chem. C 2012, 116, 23047–23052. 10.1021/jp306543q. PMC3563421 23393610

[ref16] Rahman W. N.; Bishara N.; Ackerly T.; He C. F.; Jackson P.; Wong C.; Davidson R.; Geso M. Enhancement of radiation effects by gold nanoparticles for superficial radiation therapy. Nanomed.: Nanotechnol., Biol. Med. 2009, 5, 136–142. 10.1016/j.nano.2009.01.014. 19480049

[ref17] Liu C.-J.; Wang C.-H.; Chen S.-T.; Chen H.-H.; Leng W.-H.; Chien C.-C.; Wang C.-L.; Kempson I. M.; Hwu Y.; Lai T.-C.; Hsiao M.; Yang C. S.; Chen Y. J.; Margaritondo G. Enhancement of cell radiation sensitivity by pegylated gold nanoparticles. Phys. Med. Biol. 2010, 55, 931 10.1088/0031-9155/55/4/002. 20090183

[ref18] Jain S.; Coulter J. A.; Hounsell A. R.; Butterworth K. T.; McMahon S. J.; Hyland; Muir; Dickson; Prise; Currell; O’Sullivan J. M.; Hirst D. G. Cell-specific radiosensitization by gold nanoparticles at megavoltage radiation energies. Int. J. Radiat. Oncol., Biol., Phys. 2011, 79, 531–539. 10.1016/j.ijrobp.2010.08.044. 21095075 PMC3015172

[ref19] Berbeco R. I.; Korideck H.; Ngwa W.; Kumar R.; Patel J.; Sridhar S.; Johnson S.; Price B. D.; Kimmelman A.; Makrigiorgos G. M. DNA damage enhancement from gold nanoparticles for clinical MV photon beams. Radiat. Res. 2012, 178, 604–608. 10.1667/RR3001.1. 23148509 PMC3525114

[ref20] Chattopadhyay N.; Cai Z.; Kwon Y. L.; Lechtman E.; Pignol J.-P.; Reilly R. M. Molecularly targeted gold nanoparticles enhance the radiation response of breast cancer cells and tumor xenografts to X-radiation. Breast Cancer Res. Treat. 2013, 137, 81–91. 10.1007/s10549-012-2338-4. 23160926

[ref21] Wu C.; Cai R.; Zhao T.; Wu L.; Zhang L.; Jin J.; Xu L.; Li P.; Li T.; Zhang M. Hyaluronic acid-functionalized gadolinium oxide nanoparticles for magnetic resonance imaging-guided radiotherapy of tumors. Nanoscale Res. Lett. 2020, 15, 94 10.1186/s11671-020-03318-9. 32335719 PMC7183523

[ref22] Mueller R.; Moreau M.; Yasmin-Karim S.; Protti A.; Tillement O.; Berbeco R.; Hesser J.; Ngwa W. Imaging and characterization of sustained gadolinium nanoparticle release from next generation radiotherapy biomaterial. Nanomaterials 2020, 10, 2249 10.3390/nano10112249. 33202903 PMC7697013

[ref23] Rogosnitzky M.; Branch S. Gadolinium-based contrast agent toxicity: a review of known and proposed mechanisms. BioMetals 2016, 29, 365–376. 10.1007/s10534-016-9931-7. 27053146 PMC4879157

[ref24] You Q.; Sun Q.; Yu M.; Wang J.; Wang S.; Liu L.; Cheng Y.; Wang Y.; Song Y.; Tan F.; Li N. BSA–bioinspired gadolinium hybrid-functionalized hollow gold nanoshells for NIRF/PA/CT/MR quadmodal diagnostic imaging-guided photothermal/photodynamic cancer therapy. ACS Appl. Mater. Interfaces 2017, 9, 40017–40030. 10.1021/acsami.7b11926. 29087183

[ref25] Li D.; Wen S.; Sun W.; Zhang J.; Jin D.; Peng C.; Shen M.; Shi X. One-Step Loading of Gold and Gd2O3 Nanoparticles within PEGylated Polyethylenimine for Dual Mode Computed Tomography/Magnetic Resonance Imaging of Tumors. ACS Appl. Bio Mater. 2018, 1, 221–225. 10.1021/acsabm.8b00265. 35016384

[ref26] Horiguchi Y.; Kudo S.; Nagasaki Y. Gd@ C82 metallofullerenes for neutron capture therapy—fullerene solubilization by poly (ethylene glycol)-block-poly (2-(N, N-diethylamino) ethyl methacrylate) and resultant efficacy in vitro. Sci. Technol. Adv. Mater. 2011, 12, 044607 10.1088/1468-6996/12/4/044607. 27877415 PMC5090493

[ref27] Ertas Y. N.; Jarenwattananon N. N.; Bouchard L.-S. Oxide-free gadolinium nanocrystals with large magnetic moments. Chem. Mater. 2015, 27, 5371–5376. 10.1021/acs.chemmater.5b01995.

[ref28] Ertas Y.; Bouchard L.-S. Controlled nanocrystallinity in Gd nanobowls leads to magnetization of 226 emu/g. J. Appl. Phys. 2017, 121, 093902 10.1063/1.4977511.

[ref29] Franken N. A.; Rodermond H. M.; Stap J.; Haveman J.; Van Bree C. Clonogenic assay of cells in vitro. Nat. Protoc. 2006, 1, 2315–2319. 10.1038/nprot.2006.339. 17406473

[ref30] Zhou L.; Yang T.; Wang J.; Wang Q.; Lv X.; Ke H.; Guo Z.; Shen J.; Wang Y.; Xing C.; Chen H. Size-tunable Gd2O3@ albumin nanoparticles conjugating chlorin e6 for magnetic resonance imaging-guided photo-induced therapy. Theranostics 2017, 7, 764 10.7150/thno.15757. 28255365 PMC5327648

[ref31] Sun C.; Yang H.; Yuan Y.; Tian X.; Wang L.; Guo Y.; Xu L.; Lei J.; Gao N.; Anderson G. J.; Liang X. J.; Chen C.; Zhao Y.; Nie G. Controlling assembly of paired gold clusters within apoferritin nanoreactor for in vivo kidney targeting and biomedical imaging. J. Am. Chem. Soc. 2011, 133, 8617–8624. 10.1021/ja200746p. 21542609

[ref32] Wang Y.; Wu Y.; Liu Y.; Shen J.; Lv L.; Li L.; Yang L.; Zeng J.; Wang Y.; Zhang L. W.; Li Z.; Gao M.; Chai Z. BSA-mediated synthesis of bismuth sulfide nanotheranostic agents for tumor multimodal imaging and thermoradiotherapy. Adv. Funct. Mater. 2016, 26, 5335–5344. 10.1002/adfm.201601341.

[ref33] Wang Y.; Yang T.; Ke H.; Zhu A.; Wang Y.; Wang J.; Shen J.; Liu G.; Chen C.; Zhao Y.; Chen H. Smart albumin-biomineralized nanocomposites for multimodal imaging and photothermal tumor ablation. Adv. Mater. 2015, 27, 3874–3882. 10.1002/adma.201500229. 25997571

[ref34] Shiraishi Y.; Tanaka H.; Sakamoto H.; Hayashi N.; Kofuji Y.; Ichikawa S.; Hirai T. Synthesis of Au Nanoparticles with Benzoic Acid as Reductant and Surface Stabilizer Promoted Solely by UV Light. Langmuir 2017, 33, 13797–13804. 10.1021/acs.langmuir.7b03192. 29119792

[ref35] Barreca D.; Gasparotto A.; Milanov A.; Tondello E.; Devi A.; Fischer R. A. Gd2O3 nanostructured thin films analyzed by XPS. Surf. Sci. Spectra 2007, 14, 60–67. 10.1116/11.20080703.

[ref36] Anishur Rahman A. T. M.; Majewski P.; Vasilev K. Gd2O3 nanoparticles: size-dependent nuclear magnetic resonance. Contrast Media Mol. Imaging 2013, 8, 92–95. 10.1002/cmmi.1481. 23109397

[ref37] Xie J.; Zheng Y.; Ying J. Y. Protein-directed synthesis of highly fluorescent gold nanoclusters. J. Am. Chem. Soc. 2009, 131, 888–889. 10.1021/ja806804u. 19123810

[ref38] Nosrati H.; Attari E.; Abhari F.; Barsbay M.; Ghaffarlou M.; Mousazadeh N.; Vaezi R.; Kavetskyy T.; Rezaeejam H.; Webster T. J.; et al. Complete ablation of tumors using synchronous chemoradiation with bimetallic theranostic nanoparticles. Bioact. Mater. 2022, 7, 74–84. 10.1016/j.bioactmat.2021.05.015. 34466718 PMC8379424

[ref39] Choi J.; Reipa V.; Hitchins V. M.; Goering P. L.; Malinauskas R. A. Physicochemical characterization and in V itro hemolysis evaluation of silver nanoparticles. Toxicol. Sci. 2011, 123, 133–143. 10.1093/toxsci/kfr149. 21652737

[ref40] He M.; Zhao Z.; Yin L.; Tang C.; Yin C. Hyaluronic acid coated poly (butyl cyanoacrylate) nanoparticles as anticancer drug carriers. Int. J. Pharm. 2009, 373, 165–173. 10.1016/j.ijpharm.2009.02.012. 19429302

[ref41] Sulaiman G. M.; Waheeb H. M.; Jabir M. S.; Khazaal S. H.; Dewir Y. H.; Naidoo Y. Hesperidin loaded on gold nanoparticles as a drug delivery system for a successful biocompatible, anti-cancer, anti-inflammatory and phagocytosis inducer model. Sci. Rep. 2020, 10, 9362 10.1038/s41598-020-66419-6. 32518242 PMC7283242

[ref42] Nosrati H.; Ghaffarlou M.; Salehiabar M.; Mousazadeh N.; Abhari F.; Barsbay M.; Ertas Y. N.; Rashidzadeh H.; Mohammadi A.; Nasehi L.; et al. Magnetite and bismuth sulfide Janus heterostructures as radiosensitizers for in vivo enhanced radiotherapy in breast cancer. Biomater. Adv. 2022, 140, 213090 10.1016/j.bioadv.2022.213090. 36027669

[ref43] Liu C.; Cao Y.; Cheng Y.; Wang D.; Xu T.; Su L.; Zhang X.; Dong H. An open source and reduce expenditure ROS generation strategy for chemodynamic/photodynamic synergistic therapy. Nat. Commun. 2020, 11, 1735 10.1038/s41467-020-15591-4. 32269223 PMC7142144

[ref44] Mozafari F.; Rashidzadeh H.; Ghaffarlou M.; Salehiabar M.; Ertas Y. N.; Ramazani A.; Abazari M.; Rahmati M.-A.; Javed Y.; Sharma S. K., Danafar H. ROS-Based Cancer Radiotherapy. In Harnessing Materials for X-ray Based Cancer Therapy and Imaging; Springer, 2022; pp. 265–309.

[ref45] Yadav P.; Bandyopadhyay A.; Chakraborty A.; Islam S.; Sarkar K. Enhancing the radiotherapeutic index of gamma radiation on cervical cancer cells by gold nanoparticles. Gold Bull. 2019, 52, 185–196. 10.1007/s13404-019-00260-2.

