# Supplementary material for: Enhanced In Vivo Radiotherapy of Breast Cancer Using Gadolinium Oxide and Gold Hybrid Nanoparticles
Source: ACS Appl Bio Mater. 2023 Jan 24;6(2):784–92. doi: 10.1021/acsabm.2c00965 (PMC9945098; doi:10.1021/acsabm.2c00965)
Supplement: Supplementary file 1 — mt2c00965_si_001.pdf [file mt2c00965_si_001.pdf]

Supporting Information

Enhanced in vivo radiotherapy of breast cancer using gadolinium oxide and gold hybrid nanoparticles

Hamed Nosrati[1], Marziyeh Salehiabar[1], Jalil Charmi[1], Kadir Yaray[2], Mohammadreza Ghaffarlou[3], Esra Balcioglu[4], Yavuz Nuri Ertas[1,5,6*]

[1]ERNAM—Nanotechnology Research and Application Center, Erciyes University, Kayseri, Turkey

[2]Department of Radiation Oncology, Faculty of Medicine, Erciyes University, Kayseri, Turkey

[3]Hacettepe University, Department of Chemistry, Beytepe, Ankara, Turkey

[4]Department of Histology and Embryology, Faculty of Medicine, Erciyes University, Kayseri, Turkey

[5]Department of Biomedical Engineering, Erciyes University, Kayseri, Turkey

[6]UNAM–National Nanotechnology Research Center, Bilkent University, Ankara, Turkey

*Corresponding Author :

Yavuz Nuri Ertas, Ph.D.

Email: yavuzertas@erciyes.edu.tr

ERNAM—Nanotechnology Research and Application Center, Erciyes University

UNAM–National Nanotechnology Research Center, Bilkent University, Ankara, Turkey

Kayseri 38039, Turkey

Web: www.ertaslab.com

Supporting Information

Enhanced in vivo radiotherapy of breast cancer using gadolinium oxide and gold hybrid nanoparticles

Hamed Nosrati[1], Marziyeh Salehiabar[1], Jalil Charmi[1], Kadir Yaray[2], Mohammadreza Ghaffarlou[3], Esra Balcioglu[4], Yavuz Nuri Ertas[1,5,6*]

[1]ERNAM—Nanotechnology Research and Application Center, Erciyes University, Kayseri, Turkey

[2]Department of Radiation Oncology, Faculty of Medicine, Erciyes University, Kayseri, Turkey

[3]Hacettepe University, Department of Chemistry, Beytepe, Ankara, Turkey

[4]Department of Histology and Embryology, Faculty of Medicine, Erciyes University, Kayseri, Turkey

[5]Department of Biomedical Engineering, Erciyes University, Kayseri, Turkey

[6]UNAM–National Nanotechnology Research Center, Bilkent University, Ankara, Turkey

*Corresponding Author :

Yavuz Nuri Ertas, Ph.D.

Email: yavuzertas@erciyes.edu.tr

ERNAM—Nanotechnology Research and Application Center, Erciyes University

UNAM–National Nanotechnology Research Center, Bilkent University, Ankara, Turkey

Kayseri 38039, Turkey

Web: www.ertaslab.com

Supporting figures

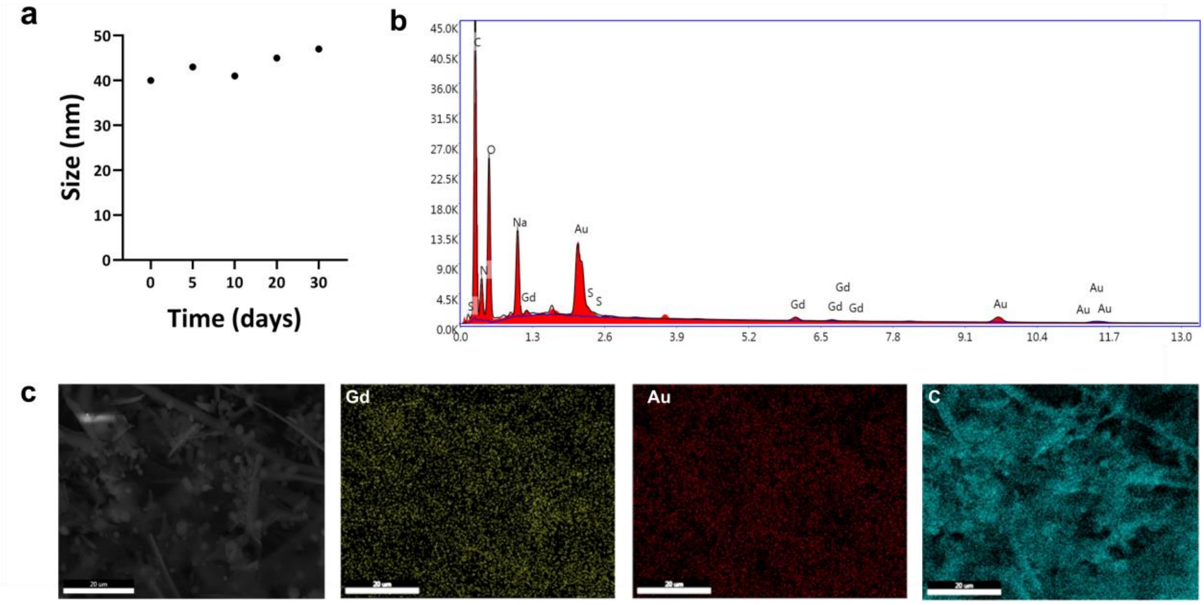

Figure S1. a) Size monitoring of Gd_2O_3@BSA-Au NPs in PBS; b) EDS spectra of Gd_2O_3@BSA-Au NPs; and (b) SEM-EDS Mapping of Gd_2O_3@BSA-Au NPs.

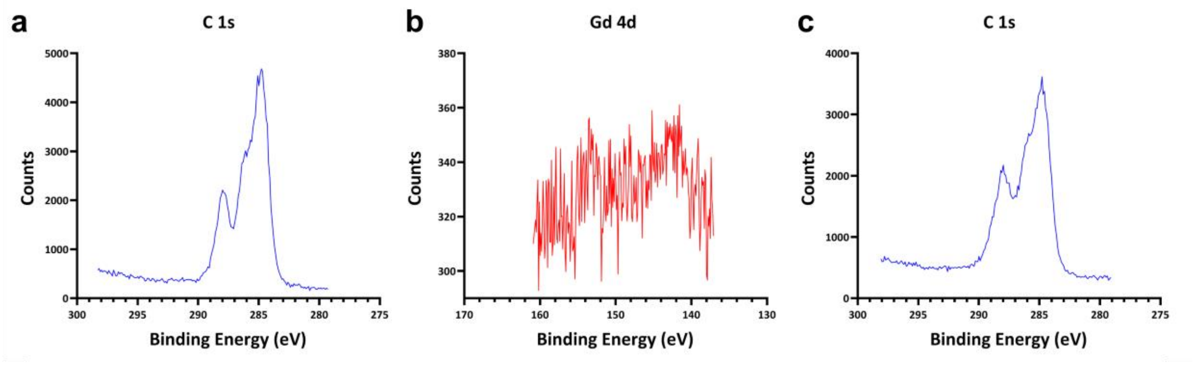

Figure S2. a) C 1S XPS spectra in Gd_2O_3@BSA; b) Gd 4d XPS spectra in Gd_2O_3@BSA-Au; c) C 1S XPS spectra in Gd_2O_3@BSA-Au.
